# Synthesis of Ordered Mesoporous Molecular Sieve-Supported Cobalt Catalyst via Organometallic Complexation for Propane Non-Oxidative Dehydrogenation

**DOI:** 10.3390/nano14131132

**Published:** 2024-06-30

**Authors:** Yanliang Zhai, Lisha Chen, Ruihan Wu, Xianggang Lu, Jun Wang, Gaolong Li, Bicheng Tang, Wei Zhang, Shaolong Zhang, Zhijun Li

**Affiliations:** 1 Provincial Key Laboratory of Polyolefin New Materials, College of Chemistry & Chemical Engineering, Northeast Petroleum University, Daqing 163318, China; zhaiyl@nepu.edu.cn (Y.Z.); 238002030231@stu.nepu.edu.cn (L.C.); jwang2020@nepu.edu.cn (J.W.);; 2 College of Chemistry and Environmental Engineering, Shenzhen University, Shenzhen 518060, China

**Keywords:** propane dehydrogenation, mesoporous molecular sieve, organometallic complexation, propylene selectivity

## Abstract

Co-based catalysts have shown great promise for propane dehydrogenation (PDH) reactions due to their merits of environmental friendliness and low cost. In this study, ordered mesoporous molecular sieve-supported CoO_x_ species (CoO_x_/Al-SBA-15 catalyst) were prepared by one-step organometallic complexation. The catalysts show worm-like morphology with regular straight-through mesoporous pores and high external specific surface area. These typical features can substantially enhance the dispersion of CoO_x_ species and mass transfer of reactants and products. Compared with the conventional impregnation method, the 10CSOC (10 wt.% Co/Al-SBA-15 prepared by the organometallic complexation method) sample presents a smaller CoO_x_ size and higher Co^2+^/Co^3+^ ratio. When applied to PDH reaction, the 10CSOC delivers higher propane conversion and propylene selectivity. Under the optimal conditions (625 °C and 4500 h^−1^), 10CSOC achieves high propane conversion (43%) and propylene selectivity (83%). This is attributed to the smaller and better dispersion of CoO_x_ nanoparticles, more suitable acid properties, and higher content of Co^2+^ species. This work paves the way for the rational design of high-performance catalysts for industrially important reactions.

## 1. Introduction

Propylene stands as a pivotal chemical raw material, with applications spanning the production of crucial chemical intermediates like polypropylene, acrylonitrile, and propylene oxide. These intermediates then serve as the foundation for synthesizing plastics, rubbers, and fibers [1,2,3]. Nevertheless, the conventional methods of propylene production, encompassing fluid catalytic cracking (FCC) and steam cracking of naphtha, have fallen short in catering to the escalating global demand for propylene [4]. The emergence of advanced shale gas and natural gas hydrate extraction techniques has augmented the accessibility and cost-effectiveness of propane, making propane dehydrogenation to propylene (PDH) a remarkably viable process [5,6]. From a thermodynamic viewpoint, the direct dehydrogenation of propane is a strong endothermic reaction, and the equilibrium conversion is about 18% and 50% at 500 °C and 600 °C, respectively. In order to achieve a higher conversion, the reaction needs to be carried out at high temperature. But at high temperature, side reactions such as C-C cracking are easy to occur, resulting in the decrease in propylene selectivity and rapid deactivation [7]. Currently, the industrialization of PDH is predominantly achieved through the Catofin process with Cr-based catalysts, and the Oleflex process with Pt-based catalysts [8,9,10,11,12]. However, further advancements in PDH technology are hindered by several challenges, including the substantial costs, expedited deactivation of Pt-based catalysts, and the environmental hazards of Cr-based catalysts [13].

Researchers have ventured into exploring diverse alternative catalysts, including metal oxides, boron and carbon-based materials [7,14,15,16,17]. Among these, Co-based catalysts have garnered increasing attention in PDH applications due to their low toxicity, cost-effectiveness, and remarkable catalytic activity [18]. However, the catalytic proficiency of these catalysts is intricately linked to the morphology and dimensions of cobalt species, which are governed by the support material and synthesis methodology [19]. Notably, the literature suggests that larger CoO_x_ nanoparticles can induce undesirable side reactions, such as coke formation and propane cracking, whereas smaller nanoparticles exhibit superior catalytic activity for propane dehydrogenation [20]. Furthermore, isolated Co^2+^ species, functioning as Lewis acidic sites, demonstrate higher catalytic performance in PDH compared to CoO_x_ nanoparticles [21,22]. Consequently, optimizing the content of isolated Co^2+^ species in Co-based catalysts through appropriate support selection and synthesis techniques poses a pressing scientific challenge. Recently, Dai et al. [22] employed a one-step hydrothermal method to synthesize a Co/Al_2_O_3_ catalyst, attributing its high propylene selectivity to the abundant presence of isolated Co^2+^ species.

In the catalysis realm, the ordered mesoporous molecular sieve Al-SBA-15 has garnered considerable attention as a catalyst support [23,24]. When compared to the traditional Al_2_O_3_ support, Al-SBA-15 exhibits several advantages, including a higher surface area, enlarged mesoporous pores, and reduced acidity. These attributes contribute significantly to enhancing the dispersion of active species, promoting the internal diffusion of reactants and products, and inhibiting cracking, isomerization, and excessive dehydrogenation reactions catalyzed by acid sites. Therefore, Al-SBA-15 emerges as a superior choice for catalyst support in the dehydrogenation of propane.

In this work, due to the high surface area and good diffusivity, highly ordered mesoporous molecular sieves Al-SBA-15 are used as supports to prepare Co/Al-SBA-15 catalysts by the organometallic complexation and impregnation methods, respectively. The cobalt species present in the catalysts are characterized and studied. The Co/Al-SBA-15 catalysts prepared by the organometallic complexation method show smaller CoO_x_ nanoparticle sizes and a higher content of Co^2+^ species. The PDH performances of the catalysts are evaluated under different reaction process parameters.

## 2. Materials and Methods

### 2.1. Materials

Polyether P123 (MW: 5800), tetraethyl orthosilicate (TEOS, 98%) and aluminium isopropoxide (99%) were purchased from Macklin Biochemical Co., Ltd. (Shanghai, China). HCl (36 wt.% in H_2_O), CoCl_2_ (99%), Co(NO_3_)_2_·6H_2_O (99%) and ethylenediamine (99%) were purchased from Aladdin Bio-Chem Technology Co., Ltd. (Shanghai, China).

### 2.2. Catalysts Preparation

#### 2.2.1. Synthesis of Ordered Mesoporous Al-SBA-15 Molecular Sieve 

Ordered mesoporous Al-SBA-15 molecular sieve was synthesized according to the following method. In a standard synthesis procedure, a specific amount of aluminum isopropoxide and 9 mL of TEOS were mixed to obtain desired Si/Al ratios of 50. After the initial mixture was formulated, it was introduced into a 10 ml aqueous HCl solution adjusted to a pH of 1.5. This mixture was vigorously agitated for a duration exceeding 3 h. Subsequently, the agitated mixture was combined with a separate solution containing 4 g of P123 dissolved in 150 mL of aqueous HCl solution, which was also set at a pH of 1.5 and maintained at 40 °C. The combined solution was further agitated for 1 h at the aforementioned temperature. Subsequently, the mixture was enclosed in Teflon bottles and allowed to react under closed conditions at 100 °C for a period of 48 h. The resulting solid product was then separated through filtration, dried at 110 °C, and ultimately calcined in air at 600 °C for a duration of 6 hours, with a controlled heating rate of 1 °C/min. This final sample was labeled as Al-SBA-15.

#### 2.2.2. Preparation of Co/Al-SBA-15 Catalyst by Organometallic Complexation Method 

Co/Al-SBA-15 catalyst was prepared by organometallic complexation method according to the following method. In a standard synthesis procedure, a specific amount of aluminum isopropoxide and 9 mL of TEOS were mixed to obtain desired Si/Al ratio of 50. Subsequently, the mixture was introduced into a 10 mL aqueous HCl solution adjusted to a pH of 1.5. After stirring vigorously for over 3 h, it was combined with a second solution containing 4 g of P123 dissolved in 150 ml of aqueous HCl solution, maintained at a pH of 1.5, and held at 40 °C. The combined mixture was agitated for an hour and designated as solution A. Next, a 50 mL ethylenediamine aqueous solution containing 10 vol.% ethylenediamine was prepared. Subsequently, various amounts of CoCl_2_ were weighed based on the desired cobalt loading (1 wt.%, 3 wt.%, 5 wt.%, 10 wt.%, and 15 wt.%). The ethylenediamine aqueous solution was then slowly added to the respective CoCl_2_ samples and agitated for 1 h, resulting in solution B. Afterward, solution B was gradually added to solution A, and the combined mixture was stirred for an additional hour. The resulting mixture was then transferred into closed Teflon bottles and allowed to react at 100 °C for 48 h. Following the reaction, the solid product was isolated via filtration and subsequently dried at 110 °C. Finally, the product was calcined in air at 600 °C for 6 h, with a heating rate of 1 °C/min. The resulting samples were labeled as 1CSOC, 3CSOC, 5CSOC, 10CSOC, and 15CSOC, based on their cobalt loading.

#### 2.2.3. Preparation of Co/Al-SBA-15 Catalyst by Impregnation Method 

Co/Al-SBA-15 catalyst was prepared by impregnation method according to the following method. A predetermined quantity of cobalt nitrate aqueous solution, corresponding to a cobalt loading of 10 wt.%, was introduced into the Al-SBA-15 molecular sieve powder utilizing an equal-volume impregnation technique. This mixture was agitated consistently to ensure uniform dispersion. Following an overnight impregnation period at ambient temperature, the mixture was subjected to drying in a 100 °C oven for 8 h. Subsequently, it underwent calcination in a muffle furnace, with a heating rate of 10 °C/min, for a duration of 6 h. The resulting sample was designated as 10CSIM.

### 2.3. Characterization

X-ray powder diffraction (XRD) patterns were acquired using a Rigaku D/MAX-2200 diffractometer, employing Cu Kα radiation (λ = 1.5406 Å) in the 2θ range of 5°–80° at a scanning rate of 2 °/min. High-resolution transmission electron microscopy (HR-TEM) analysis was carried out on an FEI TECNAI G2 F20 instrument, operating at an acceleration voltage of 200 kV.

N_2_ adsorption–desorption measurements were performed on a Micromeritics ASAP 2460 system at −196 °C. The total specific surface area was calculated by Brunauer–Emmett–Teller (BET) equation, the internal/external surface area and micropore volume were determined by the t-plot method, while the mesopore volume were determined by the BJH method.

UV-Vis spectroscopy was conducted using a Shimadzu UV-3600 spectrometer. Using BaSO_4_ as a reference, the measuring range is in the area of 300–800 nm wavelength. XPS analysis was performed on a Thermo Scientific EscaLab 250Xi instrument with Al Kα radiation; the maximum beam current is 4 μA. ICP-OES measurements were obtained from a PerkinElmer Optima 7300 V analyzer. A 50 mg sample was put into 50 ml polytetrafluoride lining, 3 ml nitric acid and 2 ml hydrofluoric acid were added, digested at 160 °C for 6 h, then volume was fixed to 50 ml with ultrapure water, diluted with 2% nitric acid and tested.

For ammonia temperature programmed desorption (NH_3_-TPD), a Xianquan TP-5076 adsorption instrument equipped with a thermal conductivity detector (TCD) was employed. A total of 0.1 g of 20–40 mesh samples were activated at 600 °C for 1 hour under N_2_ atmosphere. After cooling to 100 °C, the samples were saturated with NH_3_ for 30 min, followed by flushing with N_2_ for 30 min to remove the physically adsorbed NH_3_. Finally, the desorption of NH_3_ was carried out by heating the samples to 600 °C at a rate of 10 °C/min and the signal of the desorbed NH_3_ was recorded by TCD simultaneously. For H_2_ temperature programmed reduction (H_2_-TPR), a Xianquan TP-5080 instrument with a TCD was utilized. The sample was purged and activated at 150 °C in nitrogen atmosphere for 1 h, then cooled to 100 °C, and then mixed evenly with N_2_ with 5 vol% of H_2_ (gas flow rate of 50 mL/min), and then reduced from 100 °C at a rate of 10 °C/min to 800 °C. At the same time, the consumed H_2_ signal was analyzed and recorded by TCD.

### 2.4. Catalytic Reaction

Under atmospheric pressure, propane dehydrogenation was carried out in an 8 mm diameter quartz reactor using Co/ZSM-5 catalysts. To facilitate the PDH reaction, 200 mg of catalyst were placed in a quartz reactor, supported by quartz wool. The reactor was then heated to 550 °C under a continuous flow of N_2_ at 20 mL/min. Following a 30 mine nitrogen purge, the PDH reaction was initiated, occurring within a temperature range of 500–650 °C. The feed gas comprised a mixture of propane and nitrogen, with a propane concentration of 10 vol.% C_3_H_8_ and a gas hourly space velocity (GHSV) ranging from 1500 to 10,500 h^−1^. The GHSV is calculated as follows:

(1) $$GHSV=\frac{V_{C_{3}H_{8}}}{T\cdot V_{catalyst}}$$

where V_C3H8_ was the total volume of propane gas in feed flows, V_catalyst_ was the volume of catalyst, and T was the total reaction time of PDH.

For product analysis, an online gas chromatograph (Agilent 7890A) equipped with a flame ionization detector (FID) and HP-Plot Al_2_O_3_ capillary column (50 m × 0.53 mm, 20 μm) was utilized. The propane conversion and propylene selectivity were subsequently determined using established methodologies:

(2) $${\left[C_{3}H_{8}\right]}_{\mathrm{Conversion}}=\frac{{\left[C_{3}H_{8}\right]}_{\mathrm{In}}-{\left[C_{3}H_{8}\right]}_{\mathrm{out}}}{{\left[C_{3}H_{8}\right]}_{\mathrm{In}}}\times 100\%\;$$

(3) $${\left[C_{3}H_{6}\right]}_{\mathrm{Selectivity}}=\frac{{\left[C_{3}H_{6}\right]}_{\mathrm{out}}}{{\left[C_{3}H_{8}\right]}_{\mathrm{In}}-{\left[C_{3}H_{8}\right]}_{\mathrm{out}}}\times 100\%$$

where [C_3_H_8_]_In_ and [C_3_H_8_]_out_ were the molar contents of propane in feed and exit flows, and [C_3_H_6_]_out_ was the molar content of propylene in exit flow.

## 3. Results and Discussion

The small-angle XRD characterization shows that the Al-SBA-15 samples synthesized in this study have three diffraction peaks at 2θ = 0.9°, 1.5° and 1.8°, which are attributed to the reflection of the plane group P6mm symmetrical hexagonal structure (Figure S1) [25]. This shows that Al-SBA-15 molecular sieve has a highly ordered mesoporous structure, indicating that the Al-SBA-15 mesoporous molecular sieve has been successfully synthesized. By contrast, 10CSOC samples prepared by the organometallic complexation method and 10SI samples prepared by the impregnation method showed three diffraction peaks at 2θ = 0.9°, 1.5° and 1.8°, and the peak intensity of 10CSOC samples was higher than that of 10CSIM samples, which indicated that Co/Al-SBA-15 molecular sieves prepared by the organometallic complexation method and the impregnation method had a highly ordered mesoporous structure, indicating that the Co/Al-SBA-15 mesoporous molecular sieves were successfully synthesized (Figure S1). The mesoporous order of the samples prepared by the organometallic complexation method is higher than the samples prepared by the impregnation method.

**Figure 1:**
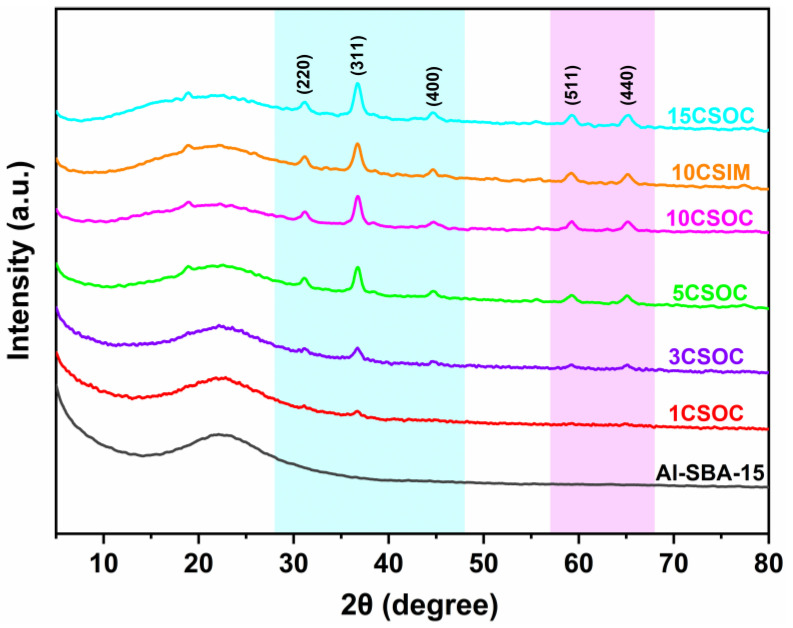
XRD patterns of different Co/Al-SBA-15 catalysts.

The wide-angle XRD spectra of the samples are shown in Figure 1. All of these Co/Al-SBA-15 samples show characteristic diffraction peaks at 2θ = 31.2°, 36.9°, 45.1°, 59.6° and 66.1°, which are attributed to the crystal planes of Co_3_O_4_ (JCPDS #43-1003) [26]. With the increase in cobalt loading, the intensity of the characteristic diffraction peaks at 2θ = 31.2°, 36.9°, 45.1°, 59.6° and 66.1° increased gradually, indicating that the content of Co_3_O_4_ crystal phase in the catalyst increased gradually. Through the characterization of small-angle XRD and wide-angle XRD, it was proved that the CoO_x_/Al-SBA-15 catalysts were successfully synthesized by the organometallic complexation method and the impregnation method.

**Figure 2:**
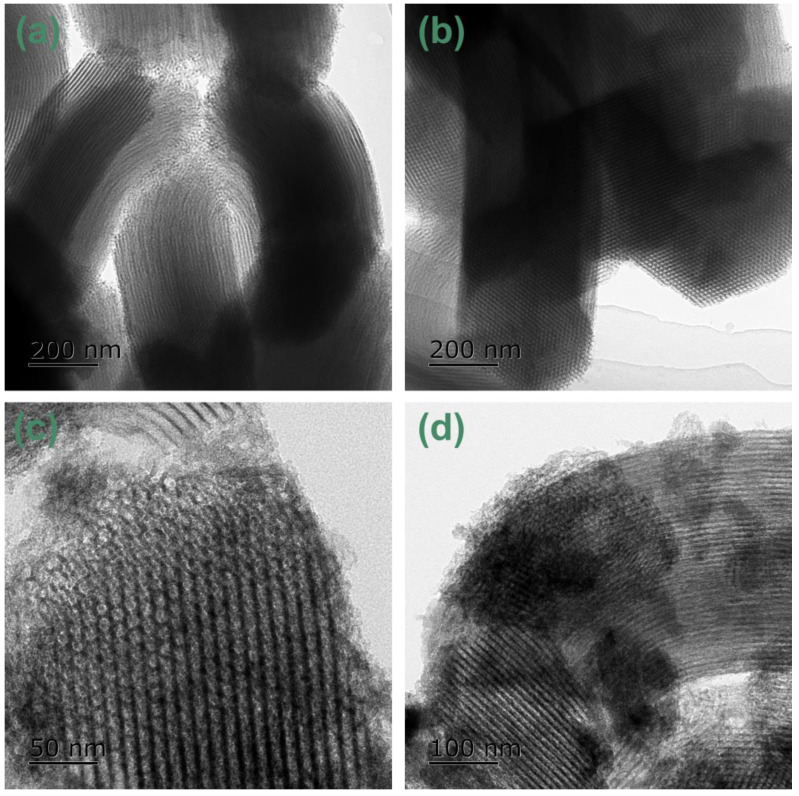
TEM images of (**a**) Al-SBA-15, (**b**) 5CSOC, (**c**) 10CSOC and (**d**) 10CSIM samples.

Figure 2 is the TEM image of the Co/Al-SBA-15 sample. All the samples showed worm-like particles with a width of about 200nm and a length of about 600nm, accompanied by highly ordered straight-through mesoporous channels. Further careful observation showed that the 10CSOC samples prepared by organometallic complexation method showed a large number of highly ordered P6mm symmetrical hexagonal mesopores, and the degree of mesoporous order was even higher than that of the 10CSIM samples prepared by the impregnation method. In addition, the sizes of the CoO_x_ nanoparticles of the 10CSOC samples prepared by the organometallic complexation method are 2–4 nm, which are small and uniformly dispersed, while those of 10CSIM samples prepared by impregnation method are 5–30 nm, with different sizes and uneven dispersion. In the process of preparing Co/Al-SBA-15 catalyst by the organometallic complexation method, the ethylenediamine ligand in ethylenediamine cobalt complex can effectively block the agglomeration of cobalt species and improve the dispersion of cobalt species, so the CoO_x_ species with high dispersion and smaller nanoparticle sizes were obtained after calcination [27]. However, for the samples prepared by impregnation method, most of the cobalt species are distributed on the outer surface of Al-SBA-15 molecular sieve, and the particle size of cobalt species is not limited, which leads to large growth and uneven size. According to Gao et al. [28], the smaller the size of cobalt oxide particles, the higher the catalytic performance of PDH. Compared with the samples prepared by precipitation method, because of the small particle size of cobalt oxide, the PDH activity and propylene selectivity can be improved.

**Figure 3:**
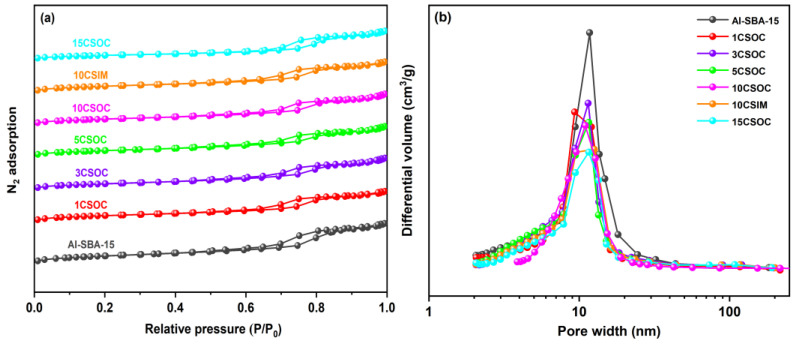
(**a**) N_2_ adsorption and desorption curves and (**b**) pore size distribution of different Co/Al-SBA-15 catalysts.

Figure 3a shows the N_2_ adsorption–desorption isotherm. Type IV isotherms with hysteresis loops were observed in all samples, indicating the existence of mesoporous [29]. Table 1 shows the texture properties and chemical composition of these samples. The Al-SBA-15 sample exhibits the highest specific surface area, reaching 736 m^2^/g, and possesses the largest mesoporous volume of 1.21 cm^3^/g. This can be attributed to the exceptional surface area and mesoporous capacity of the Al-SBA-15 molecular sieves, which feature an abundant number of highly organized and straight-running mesoporous channels. Using the zeolite as the catalyst carrier, the diffusivity of the catalyst and the catalytic performance of PDH were greatly improved. The actual cobalt content of different Co/Al-SBA-15 catalysts was determined by ICP-OES, as shown in Table 1. In the case of Co/Al-SBA-15 samples synthesized via the organometallic complexation approach, the incorporation of varying Co loadings led to a decrease in both the surface area and mesoporous volume, with an inverse correlation observed between these properties and the increasing Co content. The decrease in surface area and mesoporous volume observed in Co/Al-SBA-15 samples prepared via the organometallic complexation method can be attributed to the small size of the CoO_x_ species, which fill a significant portion of the mesoporous channels within the Al-SBA-15 molecular sieves. As Co loading increases, the occupation of these channels intensifies, causing a corresponding reduction in both surface area and mesoporous volume. Conversely, in the 10CSIM samples synthesized by the impregnation method, the migration of small CoO_x_ species into the interior of the mesoporous channels also leads to a decrease in both external area and mesoporous volume, thereby resulting in lower surface area and mesoporous volume compared to the Al-SBA-15 samples.

Figure 3b presents the BJH pore size distribution analysis of various Co/Al-SBA-15 zeolite catalysts. The figure reveals that the pore sizes of the Al-SBA-15 molecular sieves are predominantly within the 8–20 nm range. In the case of Co/Al-SBA-15 samples synthesized via the organometallic complexation method with varying cobalt loadings, the mesoporous pore size distribution of 15CSOC samples exhibits a narrowing trend as cobalt loading increases. Specifically, the pore size distribution of 15CSOC samples is confined to the 8–15 nm range. This narrowing effect can be attributed to the gradual accumulation of cobalt species within the mesoporous channels of the Al-SBA-15 molecular sieves, which, with increasing cobalt loading, results in a reduction in the size of the mesoporous pores.

The acidity properties of the catalyst, encompassing acid content and strength, can be characterized through NH_3_-TPD analysis. As evident from Table 2, the Al-SBA-15 sample stands out with the highest levels of both strong and weak acids. The origin of the strong acid sites in Al-SBA-15 zeolites lies in the attraction of positively charged H^+^ protons by the four-coordinated AlO_4_^-^ units within the molecular sieve framework. This indicates that during the one-step hydrothermal synthesis, some Al species successfully integrate into the framework structure, giving rise to these strong acid sites. However, with an incremental cobalt loading in the Co/Al-SBA-15 series, a notable trend emerges, the strong acid content gradually diminishes, while the medium acid content rises. This shift can be attributed to the interaction between the highly dispersed CoO_x_ species and the skeletal Al, leading to the formation of new medium-strong acid sites. Consequently, the amount of strong acid decreases, while the amount of medium-strong acid increases. This stability of the highly dispersed CoO_x_ species in the catalyst samples synthesized via the organometallic complexation method is further corroborated by this observation. According to Razavian et al. [30], strong acidity can promote undesirable side reactions, such as propane cracking, thereby deteriorating its dehydrogenation performance for propane to propylene conversion. Therefore, a catalyst with a low strong acid content and a moderate medium strong acid content is preferential for enhancing the catalytic efficiency of the non-oxidative dehydrogenation of propane to propylene.

**Figure 4:**
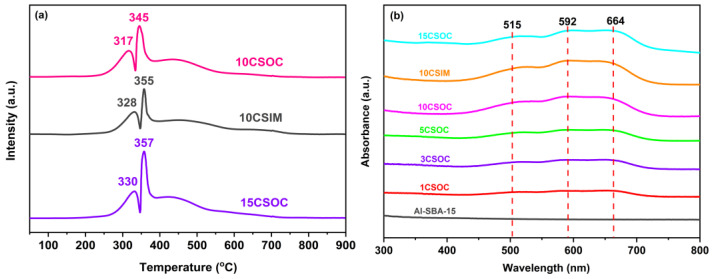
(**a**) H_2_-TPR curves and (**b**) UV-Vis spectra of different Co/Al-SBA-15 catalysts.

The TPR pattern of Co/Al-SBA-15 catalyst is shown in Figure 4a. There are two reduction peaks in 10CSOC samples at 317 °C and 345 °C. The first peak corresponds to the reduction from Co^3+^ to Co^2+^, and the second peak corresponds to the reduction from Co^2+^ to elemental Co. It shows that the cobalt species in the catalyst mainly exist in the form of Co_3_O_4_. For the 15CSOC samples also prepared by the organometallic complexation method, the two reduction peaks migrate to higher temperatures, which means that there are larger CoO_x_ species in the 15CSOC samples, which are more difficult to reduce [22,31]. In addition, compared with the 10CSOC samples prepared by the organometallic complexation method, the two reduction peak temperatures of the 10CSIM samples prepared by the impregnation method are higher. This shows that the particle size of the CoO_x_ species prepared by the metal complexation method is smaller and easier to reduce than that prepared by the impregnation method under the same loading. The experimental results are consistent with those characterized by transmission electron microscopy. Combined with the results of transmission electron microscope characterization, it can be further confirmed that compared with the 10CSIM samples prepared by the impregnation method, the 10CSOC samples prepared by organometallic complexation method have smaller nanoparticle size, better dispersion and stability. The smaller size of the CoO_x_ active species has higher catalytic activity for propane dehydrogenation, which is beneficial to improve the catalytic activity, propylene selectivity and catalytic stability of the PDH catalyst.

Figure 4b shows the UV-vis spectra of different Co/Al-SBA-15 series samples. There is no obvious absorption peak in the original molecular sieve absorption band of Al-SBA-15. For the Co/Al-SBA-15 series samples prepared by organometallic complex method, all the samples have two wide absorption bands in the 490–520 nm and 590–680 nm regions. Notably, the absorption band observed at 490–520 nm is attributed to the charge transfer transition from O^2-^→Co^2+^, while the band spanning 590–680 nm is due to the charge transfer transition from O^2-^→Co^3+^. As cobalt loading increases, the intensity of these absorption bands also intensifies [32], indicating a strengthening of the respective charge transfer transitions. The above results show that the samples prepared by the organometallic complex method with different cobalt loading contain Co^2+^ and Co^3+^, indicating that the CoO_x_ species in the catalyst mainly exist in the form of Co^2+^ and Co^3+^. There is no significant difference in the UV spectra of the 10CSIM samples prepared by the impregnation method and the 10CSOC samples prepared by the organometallic complexation method.

**Figure 5:**
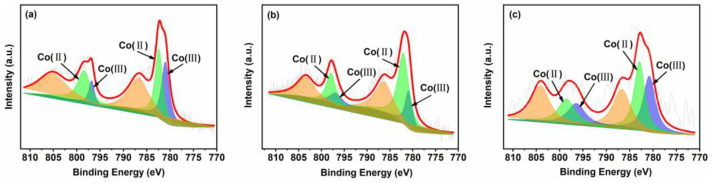
XPS spectra of (**a**) 5CSOC, (**b**) 10CSOC and (**c**) 10CSIM samples.

The Co 2p XPS spectra are shown in Figure 5. In all Co 2p spectra, there are two main peaks around 795.0–799.8 eV and 780.0–785.6 eV, which can be attributed to the Co 2p_1/2_ and Co 2p_3/2_ spin orbitals. Two parts of the Co 2p_3/2_ signal can be identified at 780.4–781.9 eV and 782.1–783.1 eV, can be attributed to Co^2+^ and Co^3+^, respectively. The Co 2p_1/2_ signal can be further divided into two parts at 795.5–796.5 eV and 797.1–798.3 eV, ascribable to Co^2+^ and Co^3+^, respectively [33]. Moreover, quantitative analyses indicate that the ratio of Co^2+^/Co^3+^ follows the order 10CSOC > 10CSIM > 5CSOC (Table S1). These results demonstrate that the CoO_x_ species of all samples contain Co^2+^ and Co^3+^ ions, which consist of Co_3_O_4_. By the studies conducted by Dai et al. [22], Co^2+^ serves as the primary catalytic active site for propane dehydrogenation. An increased concentration of Co^2+^ effectively enhances the catalytic activity, propylene selectivity, and long-term stability of the dehydrogenation process. This suggests that optimizing the Co^2+^ content in the catalyst can significantly improve the overall performance of propane dehydrogenation.

**Figure 6:**
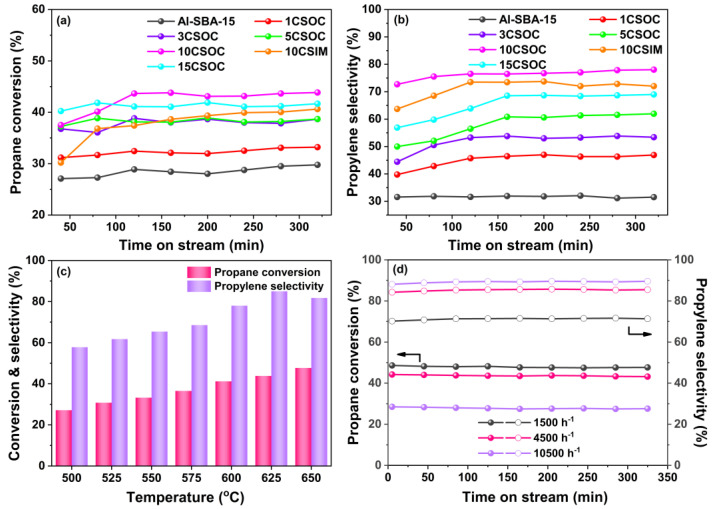
(**a**) Propane conversion and (**b**) propylene selectivity profiles of Co/Al-SBA-15 catalysts; PDH performances of 10CSOC samples at different (**c**) reaction temperatures (500–650 °C) and (**d**) GHSV (1500–10,500 h^−1^).

In Figure 6a, the outcomes of the PDH reaction are presented. Specifically, Figure 6a,b illustrate the profiles of propane conversion and propylene selectivity achieved using Co/Al-SBA-15 catalysts. These results were obtained under a reaction temperature of 600 °C and a GHSV of 4500 h^−1^. At this temperature, the thermodynamic equilibrium conversion of propane dehydrogenation is ~50% [34].

As depicted in Figure 6a,b, the highly ordered mesoporous Al-SBA-15 molecular sieve exhibits the lowest propane conversion and propylene selectivity. This suboptimal PDH performance is attributed to its primary reliance on strong acid sites for propane cracking, stemming from the absence of a dehydrogenation active center. However, as Co loading increases, the propane conversion and propylene selectivity of the Co/Al-SBA-15 catalysts exhibit an initial upward trend, followed by a decline. During the stable phase of the PDH reaction, the 10CSOC catalyst stands out, achieving a remarkable propane conversion of 44% and propylene selectivity of 78%. Notably, it sustains a propane conversion above 43% even after 5 hours, highlighting its exceptional catalytic stability. The propane conversion of 10CSOC sample is close to the thermodynamic equilibrium conversion, which is quite high. This favorable performance can be ascribed to several factors. Firstly, the Al-SBA-15 mesoporous molecular sieve support, though rich in strong acid sites conducive to propane cracking, exhibits unfavorable characteristics for PDH reactions. However, the introduction of CoO_x_ species reduces the number of strong acid sites while enhancing medium acid sites, thus enhancing PDH performance. Secondly, low Co loading leads to smaller CoO_x_ nanoparticles and higher dehydrogenation activity. As Co loading increases, the CoO_x_ species cannot be fully accommodated within the straight-through mesopores of the Al-SBA-15 zeolite, resulting in the formation of larger nanoparticles on the outer surface, ultimately degrading PDH performance. Finally, the exceptional catalytic stability of the 10CSOC catalyst is attributed to the good diffusivity and high dispersion of CoO_x_ species, facilitated by the ordered mesoporous structure of the Al-SBA-15 molecular sieve support.

As shown in Figure 6a,b, the highly ordered mesoporous Al-SBA-15 molecular sieve shows the lowest propane conversion and propylene selectivity. Due to the absence of a dehydrogenation active center, the Al-SBA-15 sample catalyzes propane cracking primarily through strong acid sites, leading to suboptimal PDH performance. However, as the Co loading increases, the propane conversion and propylene selectivity of Co/Al-SBA-15 catalysts initially rise and then decline. Notably, during the stable phase of the PDH reaction, the 10CSOC catalyst demonstrates the highest propane conversion of 44% and propylene selectivity of 78%. Additionally, it maintains a propane conversion above 43% even after 5 hours, indicating remarkable catalytic stability. These favorable outcomes can be attributed to several factors. Firstly, the Al-SBA-15 mesoporous molecular sieve support possesses a high concentration of strong acid sites, favoring propane cracking and the formation of C_1_-C_2_ hydrocarbons, which is disadvantageous for the PDH reaction. However, when CoO_x_ species are loaded onto this support, the number of strong acid sites decreases while the medium acid sites increase. As medium acid sites do not catalyze propane cracking, this enhances the PDH performance. Second, when the Co loading amount is low, the size of the CoO_x_ nanoparticles is smaller and the dehydrogenation activity is higher. With an increase in the Co loading amount, the CoO_x_ species cannot be fully accommodated in the straight-through mesoporous of the Al-SBA-15 zeolite, and some CoO_x_ species migrate to the outer surface of zeolite and form large nanoparticles, resulting in lower PDH performance. Lastly, the excellent catalytic stability of 10CSOC is attributed to the good diffusivity and high dispersion of CoO_x_ species facilitated by the ordered mesoporous structure of the Al-SBA-15 molecular sieve support.

During the stable phase of the PDH reaction, for an equivalent loading, the 10CSOC catalyst exhibits superior propane conversion and propylene selectivity compared to 10CSIM. This is because of the smaller CoO_x_ nanoparticle size and higher Co^2+^/Co^3+^ ratio of 10CSOC compared with 10CSIM. Existing research suggests that smaller CoO_x_ nanoparticles contribute to a greater PDH activity [35]. Furthermore, Co^2+^ species serve as the primary active site for PDH, and the 10CSOC catalyst, synthesized via the organometallic complexation method, contains a higher concentration of Co^2+^ species. Conversely, the lower Co^2+^ species concentration in the 10CSIM catalyst results in inferior catalytic performance for propane dehydrogenation compared to the 10CSOC catalyst prepared using the organometallic complexation approach.

As shown in Figure 6c, the PDH performance of the 10CSOC sample was evaluated at varying reaction temperatures ranging from 500 to 650 °C, with a constant propane GHSV of 4500 h^-1^. Notably, the propane conversion of 10CSOC exhibited a positive correlation with increasing reaction temperature. This enhancement is attributed to the improved catalytic activity of the active centers within the catalyst at higher temperatures, enabling the activation of a greater number of propane molecules, thus favoring the cracking reaction and enhancing propane conversion. On the other hand, the propylene selectivity of 10CSOC displayed a volcanic-shape trend with increasing reaction temperature. This is because the 10CSOC catalyst contains both an acid center and a metal center, which can simultaneously catalyze the cracking reaction and the dehydrogenation reaction. A rise in temperature favors the cracking reaction but suppresses the dehydrogenation reaction. Consequently, the interplay of these two reactions results in an initial increase in propylene selectivity, followed by a decrease. Specifically, at a reaction temperature of 625 °C, the 10CSOC catalyst achieved the optimal propylene selectivity of 85%. 

Figure 6d illustrates the PDH performance of the 10CSOC catalyst under varying GHSVs ranging from 1500 to 10,500 h^−1^, while maintaining a constant reaction temperature of 625 °C. As the propane GHSV increases, a downward trend in propane conversion is observed for the 10CSOC catalyst. Specifically, during the stable PDH reaction phase, the conversion remains around 48% at a GHSV of 1500 h^−1^, but decreases to 44% and 29% as the GHSV rises to 4500 h^−1^ and 10,500 h^−1^, respectively. This decrement is attributed to the reduced retention time of propane molecules on the 10CSOC catalyst surface at higher GHSVs, limiting the conversion rate. Therefore, reducing the GHSV to a certain extent is beneficial to improve the conversion of the PDH reaction. With an increase in the propane GHSV, the propylene selectivity of 10CSOC is raised. At a GHSV of 1500 h^−1^, the propylene selectivity reaches 71% during the stable period of the PDH reaction. When the GHSV increases to 4500 h^−1^ and 10,500 h^−1^, the selectivity increases to 86% and 90%, respectively. This trend is likely due to the shorter residence time of propane molecules at higher GHSVs, which suppresses undesired cracking reactions and favors the dehydrogenation reaction, leading to improved propylene selectivity. 

The regeneration condition of the catalyst is as follows: the catalyst was calcined in muffle furnace at 550 °C for 4 h. The performance of the twice regeneration of 10CSOC catalyst is shown in Figure S2. As can be seen from the figure, the propane conversion of the regenerated 10CSOC catalyst sample decreased slightly, and the selectivity of propylene is almost unchanged, indicating that the 10CSOC catalyst has good regeneration stability.

Drawing from these observations, we have identified the optimal operating parameters for propane dehydrogenation catalyzed by 10CSOC, namely a reaction temperature of 625 °C and a GHSV of 4500 h^−1^. Under these optimized conditions, the 10CSOC catalyst exhibits exceptional performance, achieving a propane conversion of 43% and propylene selectivity of 83%, while maintaining this high conversion rate above 43% for an extended duration of over 5 hours. This underscores the remarkable catalytic activity, selectivity, and stability of the 10CSOC catalyst for propane dehydrogenation.

## 4. Conclusions

Ordered mesoporous molecular sieves supported CoO_x_ species (CoO_x_/Al-SBA-15 catalyst) were successfully prepared by one-step organometallic complexation. The obtained catalysts show worm-like morphology with regular straight-through mesoporous pores and high external specific surface area. These typical features can substantially enhance the dispersion of CoO_x_ species and mass transfer of reactants and products. After loading Co species, strong acid amounts decrease and medium-strong acid amounts increase in varying degrees. As Co loadings rise, the external surface area and mesopore volume of xCSOC samples undergo a decline, while the propane conversion and propylene selectivity of the PDH reaction initially increase but subsequently diminish. Compared with conventional impregnation method (10CSIM), the 10CSOC sample prepared through organometallic complexation possesses a reduced CoO_x_ nanoparticle size and a heightened Co^2+^/Co^3+^ ratio. This is due to that the ethylenediamine ligand in ethylenediamine cobalt complex can effectively block the agglomeration of cobalt species and improve the dispersion of cobalt species, so the CoO_x_ species with high dispersion and smaller nanoparticles size were obtained after calcination. When applied to the PDH reaction, the 10CSOC sample exhibits superior propane conversion, propylene selectivity, and catalytic stability. This is ascribed to the appropriate pore structure and forms of metal active species. The ordered, straight-through mesoporous channels of the Al-SBA-15 support facilitate the dispersion of CoO_x_ species and the diffusion of the catalysts. Furthermore, the smaller CoO_x_ nanoparticle size and the increased content of Co^2+^ species in the 10CSOC sample enhance both its catalytic activity and stability. Under the optimized PDH conditions, namely 625 °C and a GHSV of 4500 h^−1^, the 10CSOC catalyst demonstrates high propane conversion (43%) and propylene selectivity (83%).

## Figures and Tables

**Table 1:** Texture properties of different Co/Al-SBA-15 samples.

Sample	Surface Area ^a^ (m^2^·g^−1^)			Volume ^a^ (cm^3^·g^−1^)			Co ^b^ wt.%
	BET	Exter.	Micro.	Total	Meso.	Micro.	
Al-SBA-15	736	562	174	1.26	1.21	0.05	0
1CSOC	627	464	163	1.11	1.08	0.03	0.92
3CSOC	589	433	155	0.96	0.91	0.05	2.76
5CSOC	560	410	149	0.91	0.89	0.02	4.53
10CSOC	476	341	135	0.91	0.90	0.01	9.12
10CSIM	428	318	109	0.85	0.83	0.02	8.97
15CSOC	389	285	103	0.82	0.81	0.01	13.84

^a^ Nitrogen physisorption. ^b^ ICP-OES.

**Table 2:** Acid concentration of Co/Al-SBA-15 samples determined by NH_3_-TPD.

Sample	Weak Acid (mmol/g)	Medium Acid (mmol/g)	Strong Acid (mmol/g)	Total Acid (mmol/g)
Al-SBA-15	0.73	--	0.47	1.20
1CSOC	0.71	0.17	0.46	1.34
3CSOC	0.68	0.22	0.43	1.33
5CSOC	0.63	0.25	0.36	1.24
10CSOC	0.58	0.32	0.30	1.20
10CSIM	0.59	0.31	0.32	1.22
15CSOC	0.53	0.38	0.24	1.15

## Data Availability

Data will be made available on request from the authors.

## Supplementary Materials

The following supporting information can be downloaded at: https://www.mdpi.com/article/10.3390/nano14131132/s1, Figure S1: Small-angle XRD spectra of different Co/Al-SBA-15 catalysts; Figure S2: Regeneration performance of 10CSOC sample at reaction temperatures of 625 °C and GHSV of 4500 h^−1^; Table S1: Fitting results of XPS deconvolution peaks of different Co/Al-SBA-15 catalysts.

## References

[1] Liu Z., Liu Z., Fan J., Lu W.-D., Wu F., Gao B., Sheng J., Qiu B., Wang D., Lu A.-H. (2023). Auto-accelerated dehydrogenation of alkane assisted by in-situ formed olefins over boron nitride under aerobic conditions. Nat. Commun..

[2] Foppa L., Rüther F., Geske M., Koch G., Girgsdies F., Kube P., Carey S.J., Hävecker M., Timpe O., Tarasov A.V. (2023). Data-centric heterogeneous catalysis: Identifying rules and materials genes of alkane selective oxidation. J. Am. Chem. Soc..

[3] Nakaya Y., Hirayama J., Yamazoe S., Shimizu K.-I., Furukawa S. (2020). Single-atom Pt in intermetallics as an ultrastable and selective catalyst for propane dehydrogenation. Nat. Commun..

[4] Alotaibi F.M., González-Cortés S., Alotibi M.F., Xiao T., Al-Megren H., Yang G., Edwards P.P. (2018). Enhancing the production of light olefins from heavy crude oils: Turning challenges into opportunities. Catal. Today.

[5] Wu Y., Long J., Wei S., Gao Y., Yang D., Dai Y., Yang Y. (2022). Non-oxidative propane dehydrogenation over Co/Ti-ZSM-5 catalysts: Ti species-tuned Co state and surface acidity. Microporous Mesoporous Mater..

[6] Zhao T., Shen S., Jia Y., Pao C.-W., Chen J.-L., Guo Y., Liu X., Dai S., Wang Y. (2020). Roles of niobium in the dehydrogenation of propane to propylene over a Pt/Nb-modified Al_2_O_3_ catalyst. New J. Chem..

[7] Zhao D., Tian X., Doronkin D.E., Han S., Kondratenko V.A., Grunwaldt J.-D., Perechodjuk A., Vuong T.H., Rabeah J., Eckelt R. (2021). In situ formation of ZnO_x_ species for efficient propane dehydrogenation. Nature.

[8] Motagamwala A.H., Almallahi R., Wortman J., Igenegbai V.O., Linic S. (2021). Stable and selective catalysts for propane dehydrogenation operating at thermodynamic limit. Science.

[9] Yang F., Zhang J., Shi Z., Chen J., Wang G., He J., Zhao J., Zhuo R., Wang R. (2022). Advanced design and development of catalysts in propane dehydrogenation. Nanoscale.

[10] Festa G., Contaldo P., Martino M., Meloni E., Palma V. (2023). Modeling the Selectivity of Hydrotalcite-Based Catalyst in the Propane Dehydrogenation Reaction. Ind. Eng. Chem. Res..

[11] Ma Y., Song S., Liu C., Liu L., Zhang L., Zhao Y., Wang X., Xu H., Guan Y., Jiang J. (2023). Germanium-enriched double-four-membered-ring units inducing zeolite-confined subnanometric Pt clusters for efficient propane dehydrogenation. Nat. Catal..

[12] Sun Y., Feng B., Lian Q., Xie C., Xiong J., Song W., Liu J., Wei Y. (2023). Ordered Hierarchical Porous Structure of PtSn/3DOMM-Al_2_O_3_ Catalyst for Promoting Propane Non-Oxidative Dehydrogenation. Nanomaterials.

[13] Sattler J.J., Ruiz-Martinez J., Santillan-Jimenez E., Weckhuysen B.M. (2014). Catalytic dehydrogenation of light alkanes on metals and metal oxides. Chem. Rev..

[14] Zhao Z.J., Wu T., Xiong C., Sun G., Mu R., Zeng L., Gong J. (2018). Hydroxyl-mediated non-oxidative propane dehydrogenation over VO_x_/γ-Al_2_O_3_ catalysts with improved stability. Angew. Chem. Int. Ed..

[15] Wang T.-C., Yin J.-L., Guo X.-J., Chen Y., Lang W.-Z., Guo Y.-J. (2021). Modulating the crystallinity of boron nitride for propane oxidative dehydrogenation. J. Catal..

[16] Zhu Y., Chen R., Yang Y., Liu Y., Wang X., Ji H. (2024). Low temperature catalytic activity of molybdenum promoted two-dimensional boron nitride in propane oxidative dehydrogenation reaction. Chem. Eng. J..

[17] Florou A., Bampos G., Natsi P.D., Kokka A., Panagiotopoulou P. (2023). Propylene Production via Oxidative Dehydrogenation of Propane with Carbon Dioxide over Composite M_x_O_y_-TiO_2_ Catalysts. Nanomaterials.

[18] Song S., Li J., Wu Z., Zhang P., Sun Y., Song W., Li Z., Liu J. (2022). In situ encapsulated subnanometric CoO clusters within silicalite-1 zeolite for efficient propane dehydrogenation. AlChE J..

[19] Hu Z.-P., Qin G., Han J., Zhang W., Wang N., Zheng Y., Jiang Q., Ji T., Yuan Z.-Y., Xiao J. (2022). Atomic insight into the local structure and microenvironment of isolated Co-motifs in MFI zeolite frameworks for propane dehydrogenation. J. Am. Chem. Soc..

[20] Tyo E.C., Yin C., Di Vece M., Qian Q., Kwon G., Lee S., Lee B., DeBartolo J.E., Seifert S., Winans R.E. (2012). Oxidative dehydrogenation of cyclohexane on cobalt oxide (Co_3_O_4_) nanoparticles: The effect of particle size on activity and selectivity. ACS Catal..

[21] Wu L., Ren Z., He Y., Yang M., Yu Y., Liu Y., Tan L., Tang Y. (2021). Atomically dispersed Co^2+^ sites incorporated into a silicalite-1 zeolite framework as a high-performance and coking-resistant catalyst for propane nonoxidative dehydrogenation to propylene. ACS Appl. Mater. Inter..

[22] Dai Y., Gu J., Tian S., Wu Y., Chen J., Li F., Du Y., Peng L., Ding W., Yang Y. (2020). γ-Al_2_O_3_ sheet-stabilized isolate Co^2+^ for catalytic propane dehydrogenation. J. Catal..

[23] Vu B.K., Shin E.W., Ahn I.Y., Ha J.-M., Suh D.J., Kim W.-I., Koh H.-L., Choi Y.G., Lee S.-B. (2012). The Effect of Tin–Support Interaction on Catalytic Stability over Pt–Sn/x Al–SBA-15 Catalysts for Propane Dehydrogenation. Catal. Lett..

[24] Cao L., Qiu Y., Luo S., Jiang C., Jing F. (2022). Size effect in propane dehydrogenation on PtIn/Sn-SBA-15. Mol. Catal..

[25] Kumaran G.M., Garg S., Soni K., Kumar M., Gupta J., Sharma L., Rao K.R., Dhar G.M. (2008). Synthesis and characterization of acidic properties of Al-SBA-15 materials with varying Si/Al ratios. Microporous Mesoporous Mater..

[26] Wang X., Lu S., Xu W. (2022). Synthesis of needle-like nanostructure composite electrode of Co_3_O_4_/rGO/NF for high-performance symmetric supercapacitor. Crystals.

[27] Sun Q., Wang N., Bing Q., Si R., Liu J., Bai R., Zhang P., Jia M., Yu J. (2017). Subnanometric hybrid Pd-M (OH)_2_, M= Ni, Co, clusters in zeolites as highly efficient nanocatalysts for hydrogen generation. Chem.

[28] Gao Y., Peng L., Long J., Wu Y., Dai Y., Yang Y. (2021). Hydrogen pre–reduction determined Co–silica interaction and performance of cobalt catalysts for propane dehydrogenation. Microporous Mesoporous Mater..

[29] Kleitz F., Berube F., Guillet-Nicolas R., Yang C.M., Thommes M. (2010). Probing adsorption, pore condensation, and hysteresis behavior of pure fluids in three-dimensional cubic mesoporous KIT-6 silica. J. Phys. Chem. C.

[30] Razavian M., Fatemi S. (2015). Synthesis and application of ZSM-5/SAPO-34 and SAPO-34/ZSM-5 composite systems for propylene yield enhancement in propane dehydrogenation process. Microporous Mesoporous Mater..

[31] Zhai Y., Shang Y., Zhang L., Meng X., Gong Y., Zheng L., Zhang J., Liu P. (2021). Hydrothermally modified nanosheet ZSM-5 with MnO_x_ nanoparticles and its high MTP performance. Microporous Mesoporous Mater..

[32] Bhargava R., Khan S., Ahmad N., Ansari M.M.N. Investigation of Structural, Optical and Electrical Properties of Co_3_O_4_ Nanoparticles. Proceedings of the 2nd International Conference on Condensed Matter and Applied Physics.

[33] Wang Y., Cao D., Zhao X. (2017). Heterogeneous degradation of refractory pollutants by peroxymonosulfate activated by CoO_x_-doped ordered mesoporous carbon. Chem. Eng. J..

[34] Gan Z., Dewangan N., Wang Z., Liu S., Tan X., Kawi S. (2024). Highly efficient and stable hydrogen permeable membrane reactor for propane dehydrogenation. J. Membr. Sci..

[35] Li X., Wang P., Wang H., Li C. (2018). Effects of the state of Co species in Co/Al_2_O_3_ catalysts on the catalytic performance of propane dehydrogenation. Appl. Surf. Sci..

