# Supplementary material for: Synthesis of Ordered Mesoporous Molecular Sieve-Supported Cobalt Catalyst via Organometallic Complexation for Propane Non-Oxidative Dehydrogenation
Source: Nanomaterials (Basel). 2024 Jun 30;14(13):1132. doi: 10.3390/nano14131132 (PMC11243594; doi:10.3390/nano14131132)
Supplement: Supplementary file 1 [file nanomaterials-14-01132-s001.zip › nanomaterials-3060133-supplementary.pdf]

Supplementary Material

Synthesis of Ordered Mesoporous Molecular Sieve-Supported Cobalt Catalyst via Organometallic Complexation for Propane Non-Oxidative Dehydrogenation

Yanliang Zhai [1], Lisha Chen [1], Ruihan Wu [1], Xianggang Lu [1], Jun Wang [1], Gaolong Li [1], Bicheng Tang [1], Wei Zhang [1], Shaolong Zhang [2] and Zhijun Li [1,*]

[1] Provincial Key Laboratory of Polyolefin New Materials, College of Chemistry & Chemical Engineering, Northeast Petroleum University, Daqing 163318, China; zhaiyl@nepu.edu.cn (Y.Z.)

[2] College of Chemistry and Environmental Engineering, Shenzhen University, Shenzhen 518060, China

* Correspondence: zhijun.li@queensu.ca

Supplementary Figures

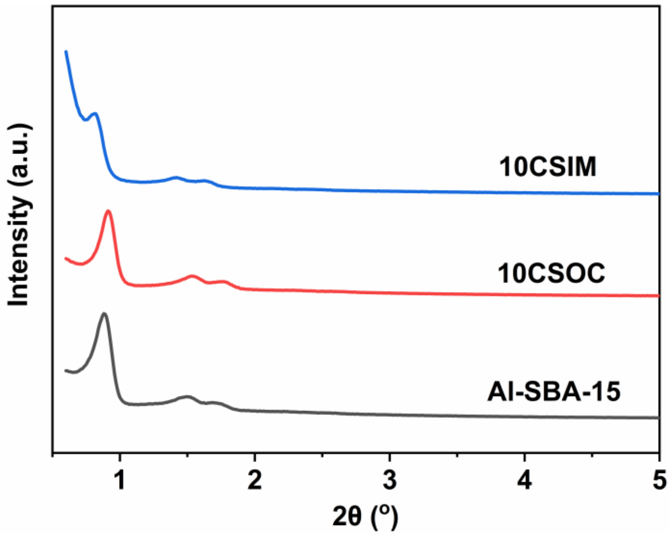

Figure S1. Small-angle XRD spectra of different Co/Al-SBA-15 catalysts

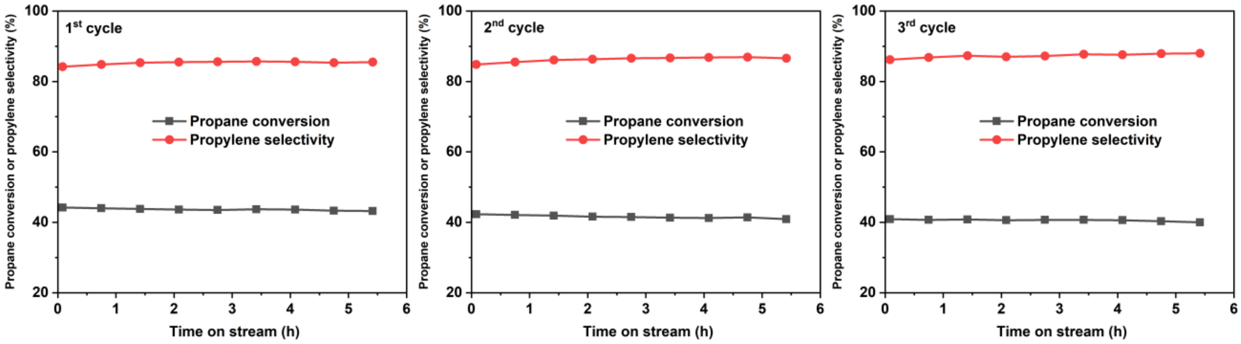

Figure S2. Regeneration performance of 10CSOC sample at reaction temperatures of 625°C and GHSV of 4500 h^{-1}

Table S1. Deconvolution peak fitting results of XPS spectra of different samples

Sample	Co^{2+}/Co^{3+} ratio
5CSOC	53/47
10CSOC	71/29
10CSIM	63/37
